# Systemic Inflammation Is Associated With Future Risk of Fatal Infection: An Observational Cohort Study

**DOI:** 10.1093/infdis/jiac186

**Published:** 2022-05-10

**Authors:** Michael Drozd, Mar Pujades-Rodriguez, Ann W Morgan, Patrick J Lillie, Klaus K Witte, Mark T Kearney, Richard M Cubbon

**Affiliations:** Leeds Institute of Cardiovascular and Metabolic Medicine, The University of Leeds, Leeds, United Kingdom; Leeds Institute of Health Sciences, School of Medicine, The University of Leeds, Leeds, United Kingdom; Leeds Institute of Cardiovascular and Metabolic Medicine, The University of Leeds, Leeds, United Kingdom; NIHR Leeds Biomedical Research Centre and NIHR Leeds Medtech and In vitro Diagnostics Co-operative, Leeds Teaching Hospitals NHS Trust, Leeds, United Kingdom; Department of Infection, Castle Hill Hospital, Hull University Hospitals NHS Trust, Kingston Upon Hull, United Kingdom; Medical Clinic 1, University Hospital Aachen, RWTH, Aachen, Germany; Leeds Institute of Cardiovascular and Metabolic Medicine, The University of Leeds, Leeds, United Kingdom; Leeds Institute of Cardiovascular and Metabolic Medicine, The University of Leeds, Leeds, United Kingdom

**Keywords:** infection, inflammation, mortality, C-reactive protein

## Abstract

**Background:**

Many diseases are associated with chronic inflammation, resulting in widening application of anti-inflammatory therapies. Although they are effective as disease-modifying agents, these anti-inflammatory therapies increase the risk of serious infection; however, it remains unknown whether chronic systemic inflammation per se is also associated with fatal infection.

**Methods:**

Using serum C-reactive protein (CRP) data from 461 052 UK Biobank participants, we defined incidence rate ratios (IRRs) for death from infection, cardiovascular disease, or other causes and adjusted for comorbidities and the use of anti-inflammatory therapies.

**Results:**

Systemic inflammation, defined as CRP ≥2 mg/L, was common in all comorbidities considered. After adjusting for confounding factors, systemic inflammation was associated with a higher IRR point estimate for infection death (1.70; 95% confidence interval [CI], 1.51–1.92) than cardiovascular (1.48; CI, 1.40–1.57) or other death (1.41; CI, 1.37–1.45), although CIs overlapped. C-reactive protein thresholds of ≥5 and ≥10 mg/L yielded similar findings, as did analyses in people with ≥2, but not <2, comorbidities.

**Conclusions:**

Systemic inflammation per se identifies people at increased risk of infection death, potentially contributing to the observed risks of anti-inflammatory therapies in clinical trials. In future clinical trials of anti-inflammatory therapies, researchers should carefully consider risks and benefits in target populations, guided by research into mechanisms of infection risk.

## Teaser

Systemic inflammation assessed using C-reactive protein is common in many chronic diseases and is associated with increased long-term risk of fatal infection. This may contribute to the observed risks of fatal infection in recipients of anti-inflammatory therapies.

Inflammation is a common pathological factor in many chronic diseases including atherosclerosis, arthritis, chronic lung disease, cancer, diabetes, and obesity [1–5]; moreover, it is more common as multimorbidity accrues [6–8]. The success of anti-inflammatory therapies as disease-modifying agents for inflammatory rheumatological, dermatological, and gastrointestinal disorders has recently prompted phase 3 clinical trials in the context of atherosclerosis [9, 10]. There is also hope that inflammation could represent a novel therapeutic target in diseases ranging from heart failure to cancer to depression [11–13]. However, canakinumab and colchicine failed to improve overall survival in people with advanced atherosclerosis, in spite of substantially reducing cardiovascular events, probably because of the increased risk of fatal infections [9, 14]. This highlights the complexity of therapeutic modulation and suggests that future approaches will require nuance [15], perhaps informed by experience from more established indications for anti-inflammatory therapy [16]. However, in spite of a wealth of experience in therapeutically targeting inflammation, it remains unclear whether systemic inflammation per se is a risk factor for serious infection, perhaps by indicating individuals more likely to mount abnormal immune responses to pathogens. We set out to answer this question using the UK Biobank (UKB) cohort study, which provides detailed phenotyping of approximately 500 000 adults, including large numbers with diverse chronic inflammatory morbidities. Since clinical trials of anti-inflammatory therapies used serum C-reactive protein (CRP) ≥2 mg/L to include people with systemic inflammation [9, 17], we used this threshold to define systemic inflammation in our analysis of UKB. Our primary objective was to define associations between CRP ≥2 mg/L and the risk of death from infection, cardiovascular disease, or other causes, including stratification by chronic diseases previously shown to be associated with increased risk of infection death [18]. We hypothesized (1) that CRP ≥2 mg/L is associated with greater relative risk of death from infection than cardiovascular or other causes and (2) that this would be observed across chronic disease strata.

## METHODS

### UK Biobank Cohort

The UKB is a population-based prospective cohort study that consists of 502 505 people aged between 37 and 73 years. The resource was developed using United Kingdom (UK) Government and biomedical research charity funding to improve understanding of disease and is an open access resource for all bona fide researchers. Full details of the study design and conduct are available from the UKB website (https://www.ukbiobank.ac.uk). Participants were recruited between 2006 and 2010 by approaching all adults living within 40 kilometers of 22 assessment centers across England, Scotland, and Wales. Detailed analysis of differences between the UK population and UK Biobank cohort is provided by Fry et al [19]. In brief, participation rates were higher in females than males (6.4% vs 5.1%), older people (9% in those aged ≥60 years and 3% in those aged 40–44 years), and in less socioeconomically deprived areas (8.3% among persons from the least deprived areas and 3.1% among persons from the most deprived areas). There was also possible underrepresentation of nonwhite ethnic groups. When compared with nationally representative data, Fry et al [19] noted lower rates of self-reported disease and lower all-cause mortality in UK Biobank participants, overall consistent with a healthy volunteer effect. Although the cohort is not representative of the whole UK population relating to socioeconomic deprivation (SED), some noncommunicable diseases and ethnic minorities, it allows assessment of exposure-disease relationships [19]. Baseline biological measurements were recorded, and participants completed a touchscreen and nurse-led questionnaire, as described elsewhere [20]. The UKB received ethical approval from the NHS Research Ethics Service (11/NW/0382); we conducted this analysis under application number 59585. All participants provided written informed consent.

### Definitions of Systemic Inflammation and Study Covariates

Systemic inflammation was defined using serum CRP data generated with a high sensitivity immunoturbidimetric assay (AU5800; Beckman Coulter). The UKB collected CRP data at study enrollment from 468 528 participants. Our primary analyses defined systemic inflammation as CRP ≥2 mg/L, based upon prior clinical trials targeting anti-inflammatory agents to people above this threshold [9, 17]. Potential confounding factors considered in the adjusted analyses were age, sex, ethnicity, SED, smoking status, comorbidity, and anti-inflammatory medical therapy, all determined at study recruitment. Ethnicity was participant-classified within UKB-defined categories of white, mixed, Asian or British Asian, black or British black, Chinese, or other ethnic group; due to the small number of people (and deaths) in each minority (non-white) ethnic group, these were pooled as “non-white ethnicity”. Smoking status was self-reported as never, previous, or current at the point of recruitment. Socioeconomic deprivation was measured using the Townsend score, an area-based deprivation index, and categorized into quintiles. Obesity was classified using the World Health Organization’s categorization according to body mass index: class 1 (30.0–34.9 kg/m^2^), class 2 (35.0–39.9 kg/m^2^), class 3 (≥40 kg/m^2^). Self-reported medical disorders recorded solely at study recruitment during face-to-face interview with a nurse were used to classify morbidities (described in Supplementary Table 1). We used clinical consensus before our analyses to select a range of morbidities that represent a broad spectrum of common disease groups: hypertension, chronic heart disease (ischemic heart disease and heart failure), chronic respiratory disease, diabetes, prior cancer, chronic liver disease, chronic kidney disease, prior stroke or transient ischemic attack (TIA), other neurological disease, psychiatric disease, and chronic inflammatory and autoimmune rheumatic disease [18]. The number of these morbidities was calculated for each participant as an index of multimorbidity. Self-reported use of nonsteroidal anti-inflammatory drugs (NSAID) or immunosuppressive agents (including disease-modifying antirheumatic drugs and oral glucocorticoids) was assessed at study enrollment as described in Supplementary Table 2. Missing data for comorbidities (*n* = 863), body mass index (*n* = 3106), smoking (*n* = 2949), ethnicity (*n* = 2777), and SED (*n* = 624) and loss to follow-up or withdrawal of consent (*n* = 1343) resulted in exclusion of 7476 participants from our analyses (some participants with more than 1 variable missing), resulting in a study cohort of 461 052 participants.

### Definition of Outcomes

Mortality information provided by UKB was derived from linked national death registry data from NHS Digital for participants in England and Wales and from the NHS Central Register, part of the National Records of Scotland, for participants in Scotland. In the present analysis, we censored follow-up and only considered deaths until December 31, 2019 to ensure this was before the first reported case of coronavirus disease 2019 in the United Kingdom [21]. As we have previously described, deaths were classified using *International Classification of Diseases, Tenth Revision* codes for the main cause of death as infection-related [18], cardiovascular [22], or other causes; specific codes are described in Supplementary Table 3. Infection death was our primary study outcome. Cardiovascular death was a secondary outcome given the wealth of data causally linking systemic inflammation to adverse cardiovascular outcomes [1, 9, 10, 15].

### Statistical Analysis

Continuous variables are presented as mean (standard deviation) or median (interquartile range [IQR]) if nonnormally distributed, and categorical variables are presented as number (percentage). Characteristics of participants with and without systemic inflammation were not formally statistically compared because these are descriptive data, rather than pertaining to a tested hypothesis. Adjusted cause-specific mortality incidence rate ratios (IRRs) were estimated using Poisson regression with exposure time modeled. Time-varying covariates were not used, and the calendar year of recruitment was not included in models due to the narrow recruitment era. Unless specified otherwise, models were adjusted for all of the following covariates: age, sex, SED, smoking status, obesity, hypertension, chronic heart disease, chronic respiratory disease, diabetes, cancer, chronic liver disease, chronic kidney disease, prior stroke/TIA, other neurological disease, psychiatric disorder, autoimmune rheumatological disease, NSAID, and immunosuppressive agent use. C-reactive protein was dichotomized as <2 or ≥2 mg/L in our primary analyses because this threshold has been applied in clinical trials of anti-inflammatory therapy [9, 14]. When addressing multimorbidity, we separately modeled the number of comorbidities (ie, number of comorbidities present at baseline, among those considered for adjustment, categorized as 1, 2, and 3 or more, because few participants had 4 or more) in place of the individual comorbidity variables (obesity, hypertension, chronic heart disease, chronic respiratory disease, diabetes, cancer, chronic liver disease, chronic kidney disease, prior stroke/TIA, other neurological disease, psychiatric disorder, and rheumatological disease). When stratifying the population by specific morbidities, or the number of comorbidities, the stratifying factor was excluded from the model. Correlation matrices of Poisson model’s coefficients were used to confirm absence of correlation between covariates (defined as >0.3 or <−0.3). As previously described [18], age was modeled using restricted cubic splines with 5 knots for infection death analyses and 4 knots for noninfection death analyses, because these provided the best fit as assessed by the Akaike and the Bayesian criterion (models including categorical, linear, cubic splines with 3, 4, and 5 knots, and first- and second-degree fractional polynomials were compared). Because the proportion of participants with missing covariate data is modest (1.6%), we did not impute missing data. Secondary analyses included the following: (1) assessment of age/sex adjusted, age/sex/sociodemographic factor/comorbidity adjusted and fully adjusted models; and (2) subgroup analyses stratified by specific disease states or multimorbidity categories. Sensitivity analyses included the following: (1) assessment of alternate CRP thresholds of ≥5 mg/L and ≥10 mg/L; and (2) exclusion of participants who died during the first 6 months of follow-up, to reduce bias from reverse causality because elevated CRP could denote acute infection. All tests were 2-sided and statistical significance was defined as *P* < .05. All statistical analyses were performed using Stata/MP (version 16.1; StataCorp LP, College Station, TX).

## RESULTS

**Figure 1.**
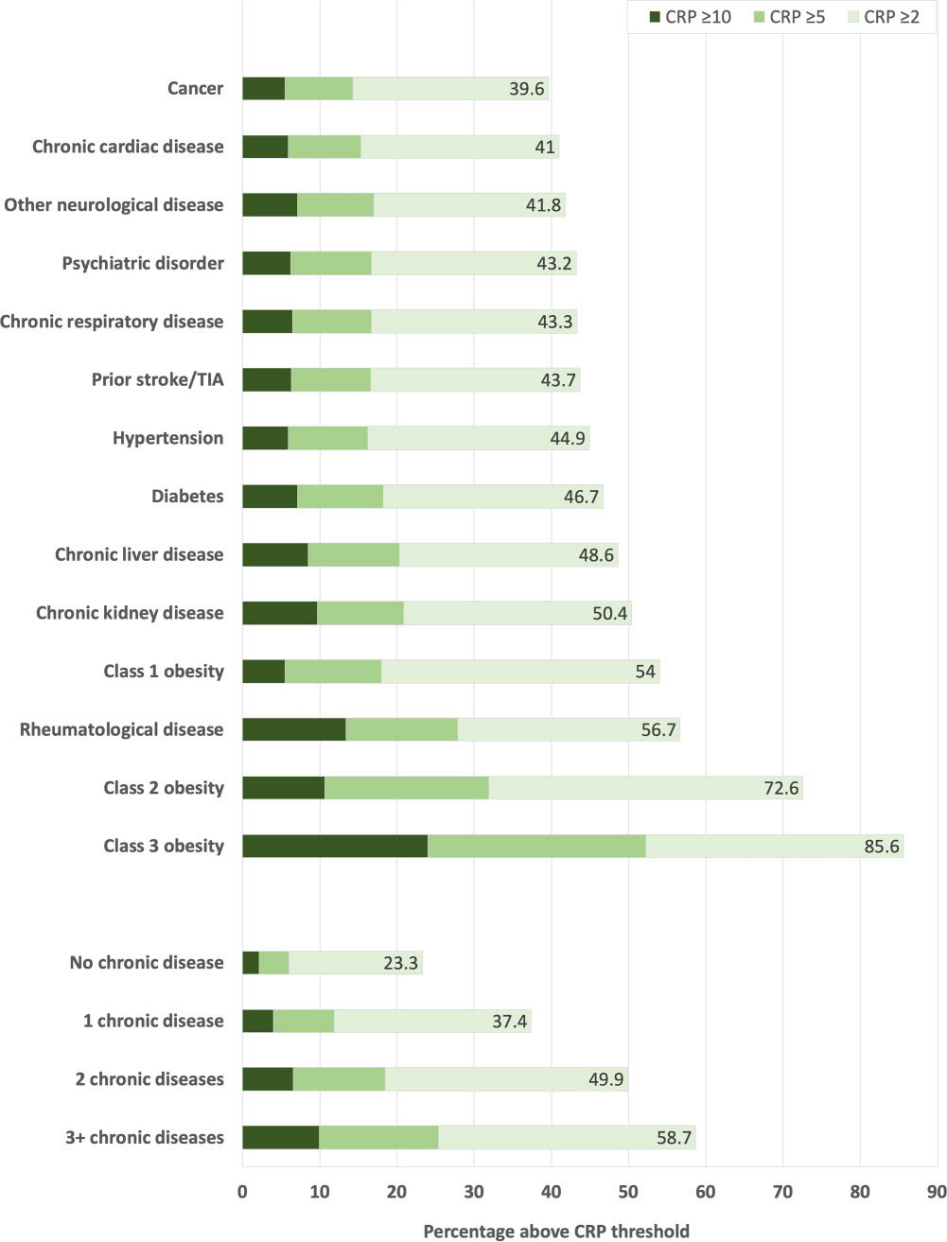
Prevalence of elevated C-reactive protein (CRP) in chronic morbidity or multimorbidity groups. Stacked bar chart illustrating the percentage of people in specified chronic disease and multimorbidity groups with CRP ≥2 mg/L, ≥5 mg/L, and ≥10 mg/L. TIA, transient ischemic attack.

Within a study population of 461 052 people, 35.2% (*n* = 162 419) had serum CRP ≥2 mg/L (characteristics of participants without CRP data are presented in Supplementary Table 4). In relation to people with CRP <2 mg/L, a higher proportion of those with CRP ≥2 mg/L were older, female, socioeconomically deprived, current smokers, and multimorbid (Table 1). Similar observations resulted from analyses of people with CRP ≥5 mg/L (11.6%; *n* = 53 468) and ≥10 mg/L (4.1%; *n* = 19 024), as shown in Supplementary Tables 5 and 6. It is notable that CRP ≥2 mg/L was highly prevalent in all of the chronic diseases studied, ranging from 39.6% of people with cancer to 85.6% of people with class 3 obesity, and was more prevalent in people with greater multimorbidity (Figure 1).

**Table 1.** Characteristics of People With CRP <2 mg/L and ≥2 mg/L

Characteristics	CRP < 2 *n* = 298 633	CRP ≥2 *n* = 162 419
Age (Years)		
<45	33 864 (11.3)	13 466 (8.3)
45 to <50	42 303 (14.2)	18 272 (11.2)
50 to <55	46 603 (15.6)	23 418 (14.4)
55 to <60	54 047 (18.1)	29 363 (18.1)
60 to <65	69 635 (23.3)	42 099 (25.9)
65+	52 181 (17.5)	35 801 (22.0)
Sex		
Female	157 308 (52.7)	93 127 (57.3)
Male	141 325 (47.3)	69 292 (42.7)
Ethnicity		
White	283 469 (94.9)	153 602 (94.6)
Non-white	15 164 (5.1)	8817 (5.4)
SED Quintile		
1 (least deprived)	63 123 (21.1)	29 166 (18.0)
2	61 832 (20.7)	30 302 (18.7)
3	60 578 (20.3)	31 641 (19.5)
4	58 928 (19.7)	33 273 (20.5)
5 (most deprived)	54 172 (18.1)	38 037 (23.4)
Smoking		
Never	171 359 (57.4)	81 086 (49.9)
Former	100 953 (33.8)	59 200 (36.4)
Current	26 321 (8.8)	22 133 (13.6)
Obesity		
Not obese	253 639 (84.9)	94 204 (58)
Class 1	37 404 (12.5)	43 938 (27.1)
Class 2	6312 (2.1)	16 703 (10.3)
Class 3	1278 (0.4)	7574 (4.7)
Hypertension	67 259 (22.5)	5842 (33.8)
Chronic cardiac disease	12 864 (4.3)	8933 (5.5)
Chronic respiratory disease	33 773 (11.3)	25 814 (15.9)
Diabetes	12 259 (4.1)	10 733 (6.6)
Cancer	22 846 (7.7)	14 975 (9.2)
Chronic liver disease	464 (0.2)	439 (0.3)
Chronic kidney disease	583 (0.2)	592 (0.4)
Prior stroke/TIA	4496 (1.5)	3483 (2.1)
Other neurological disease	3541 (1.2)	2547 (1.6)
Psychiatric disorder	15 755 (5.3)	12 004 (7.4)
Rheumatological disease	4501 (1.5)	5894 (3.6)
NSAID use	44 525 (14.9)	30 648 (18.9)
Immunosuppressant use	5609 (1.9)	6572 (4.0)
Number of Chronic Diseases		
0	149 909 (50.2)	45 502 (28.0)
1	94 988 (31.8)	56 652 (34.9)
2	37 770 (12.6)	37 566 (23.1)
3+	15 966 (5.3)	22 699 (14.0)

Abbreviations: CRP, C-reactive protein; NSAID, nonsteroidal anti-inflammatory drug; SED, socioeconomic deprivation; TIA, transient ischemic attack.

**Figure 2.**
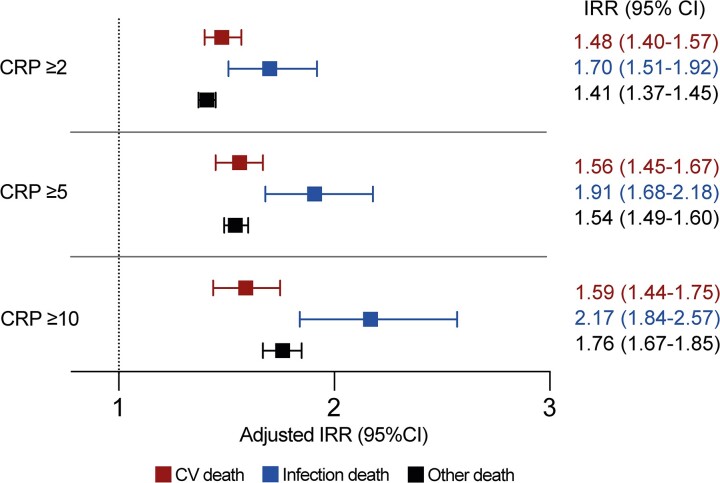
Adjusted incidence rate ratios (IRR) of cause-specific mortality according to C-reactive protein (CRP) category. Forest plot illustrating adjusted IRR and their 95% confidence intervals (CI) for specified modes of death in people with CRP ≥2 mg/L, ≥5 mg/L, and ≥10 mg/L (vs below these thresholds). The adjusted model includes the following factors in addition to CRP categories: age, sex, socioeconomic deprivation, smoking status, obesity, hypertension, chronic heart disease, chronic respiratory disease, diabetes, cancer, chronic liver disease, chronic kidney disease, prior stroke/transient ischemic attack, other neurological disease, psychiatric disorder, autoimmune rheumatological disease, self-reported nonsteroidal anti-inflammatory drug prescription, and self-reported immunosuppressive agent prescription. CV, cardiovascular.

After 4 927 012 person-years of follow-up (median 10.9 [IQR, 10.1–11.6] years per participant), 25 619 deaths (5.6% of participants) occurred. Of these, 1274 (5.0%) were attributed to infection, 5202 (20.3%) were attributed to cardiovascular events, and 19 143 (74.7%) were attributed to other causes. The IRRs for the association between CRP ≥2 mg/L and these 3 categories of death are shown in Figure 2. Results from secondary analyses presenting IRRs from unadjusted, age/sex adjusted, and other models are also presented in Supplementary Table 7. To exclude the possibility that deaths occurring early during follow-up were related to undiagnosed acute infection at the time of enrollment, we repeated analyses after excluding all deaths during the first 6 months and derived similar IRRs (Supplementary Table 8). These data illustrate that CRP ≥2 mg/L is associated with increased risk of the 3 categories of death. Point estimates for relative risk of infection death were higher than for cardiovascular or other death, although some CIs overlapped. Sensitivity analyses using higher CRP thresholds of ≥5 mg/L and ≥10 mg/L broadly yield the same conclusion, although differences in point estimates of relative risk between infection death and cardiovascular or other death were higher than for the ≥2 mg/L threshold (Figure 2). However, in spite of higher relative risk of infection death, it is important to emphasize that the absolute rate of infection death was lower than those of cardiovascular and other death, even in people with CRP ≥10 mg/L (Supplementary Table 9).

**Figure 3.**
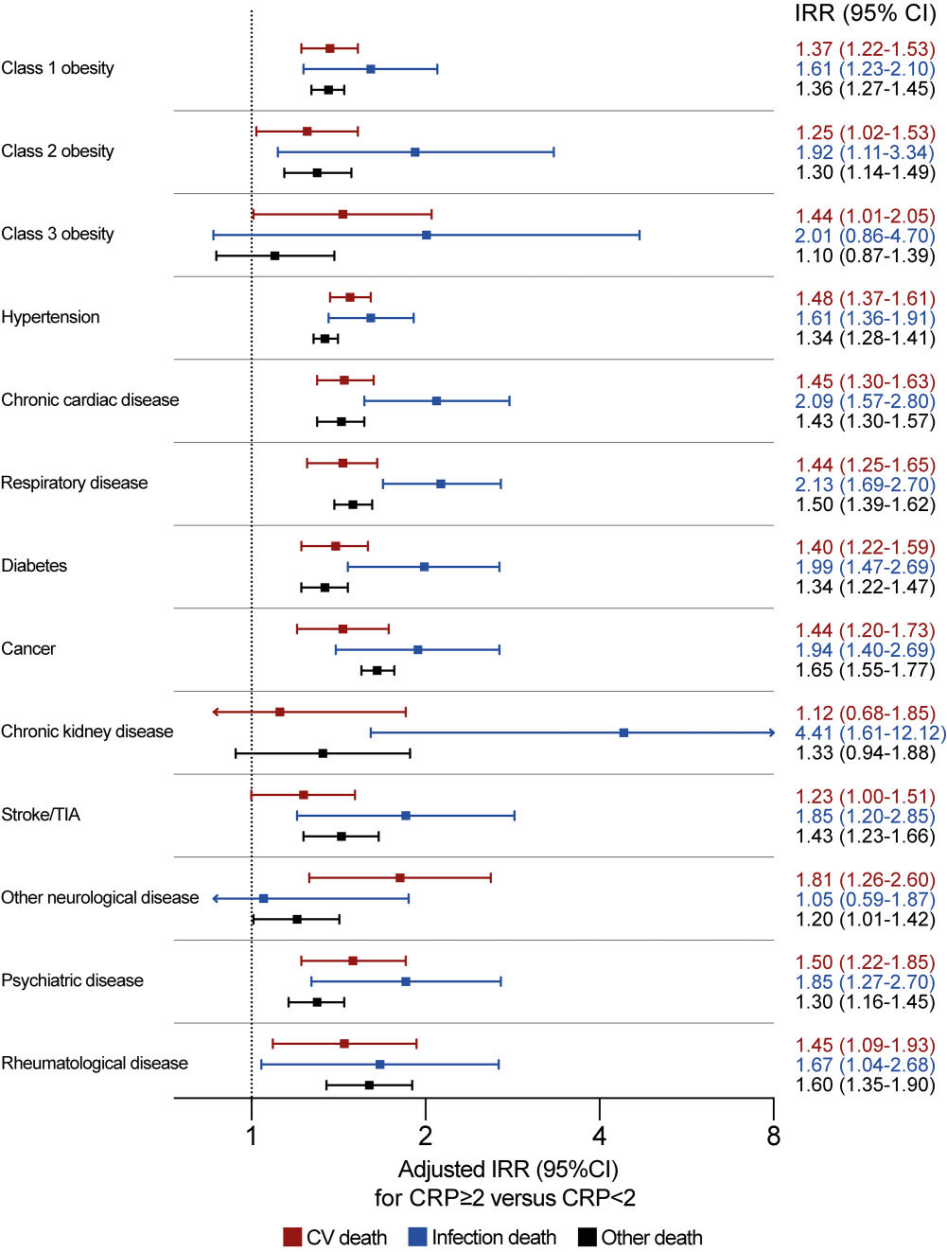
Adjusted incidence rate ratios (IRR) of cause-specific mortality in people with C-reactive protein (CRP) ≥2 mg/L stratified by chronic disease group. Forest plot illustrating adjusted IRR and their 95% confidence intervals (CI) for specified modes of death in people with CRP ≥2 mg/L vs CRP <2 mgL stratified by chronic disease group. The adjusted model includes the following factors in addition to CRP status within chronic disease group strata: age, sex, socioeconomic deprivation, smoking status, comorbidity beyond defined strata (including obesity, hypertension, chronic heart disease, chronic respiratory disease, diabetes, cancer, chronic liver disease, chronic kidney disease, prior stroke/transient ischemic attack, other neurological disease, psychiatric disorder, and autoimmune rheumatological disease), self-reported nonsteroidal anti-inflammatory drug prescription, and self-reported immunosuppressive agent prescription. CV, cardiovascular.

**Figure 4.**
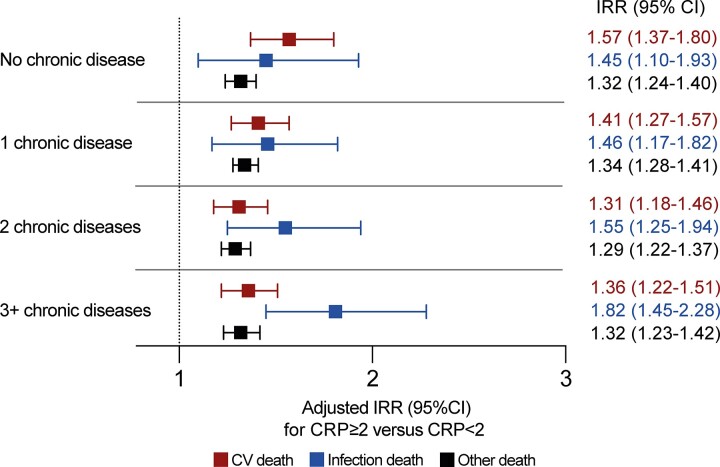
Adjusted incidence rate ratios (IRR) of cause-specific mortality in people with C-reactive protein (CRP) ≥2 mg/L stratified by number of morbidities. Forest plot illustrating adjusted IRR and their 95% confidence intervals (CI) for specified modes of death in people with CRP ≥2 mg/L vs CRP <2 mgL stratified by the extent of multimorbidity. The adjusted model includes the following factors in addition to CRP status within multimorbidity strata: age, sex, socioeconomic deprivation, smoking status, self-reported nonsteroidal anti-inflammatory drug prescription, and self-reported immunosuppressive agent prescription. CV, cardiovascular.

We also assessed whether the association between systemic inflammation and cause-specific mortality was consistent in subgroups with specific morbidities or accumulating multimorbidity. Again, CRP ≥2 mg/L was associated with increased risk of all categories of death, but nominally higher IRRs were observed for infection death than cardiovascular or other death in all morbidities except “other neurological diseases” (Figure 3). The same conclusion was reached irrespective of the number of comorbid diseases, although the IRR for infection death was nominally lower than that for cardiovascular death in people without disease (Figure 4). Sensitivity analyses applying higher CRP thresholds of ≥5 mg/L and ≥10 mg/L yielded similar conclusions (Supplementary Tables 10–13).

## DISCUSSION

Our analysis provides a novel exploration of the association between systemic inflammation and infection death and has important implications for future research and clinical practice. We show that elevated CRP is highly prevalent in many morbidities and is associated with a greater relative risk of infection death than cardiovascular or other causes of death, irrespective of the CRP threshold chosen. This observation was consistent in stratified analyses across the vast majority of diseases we studied, suggesting that it is broadly relevant to people with diverse diseases or combinations of diseases. In the context of broadening clinical use of anti-inflammatory therapies, our data caution that people identified as candidates based on elevated CRP are already predisposed to fatal infection before initiating treatment. This suggests that careful balancing of risks and benefits of such therapies is essential, which is likely to require greater understanding of how perturbed inflammation contributes to chronic diseases to personalize therapy.

Our findings may be particularly pertinent to recent clinical trials, which targeted canakinumab or colchicine to people with advanced atherosclerotic cardiovascular disease [9, 10]. Inflammation is a key driver of atherosclerosis [1, 15], and these trials demonstrated clinically important reductions in adverse cardiovascular events, yet without improving overall mortality [9, 10]. Serious infection events are also substantially increased by these agents [9, 14], which may underpin their failure to improve overall survival in people with advanced atherosclerosis; this represents a major hurdle to routine clinical adoption. Although these trials only recruited people after myocardial infarction, data from people with other cardiovascular diseases demonstrate that adverse infection events are frequent causes of death [22–25], suggesting that safely targeting inflammation will be challenging. However, in our study, although we consistently observed that elevated CRP was associated with a greater relative risk of infection than cardiovascular death, absolute rates of infection death were still appreciably lower, suggesting that safer anti-inflammatory approaches could offer overall benefit. Indeed, the targeting of Rosuvastatin to people at high cardiovascular risk with CRP ≥2 mg/L was shown to reduce inflammation, along with improving cardiovascular events “and” all-cause mortality [17].

Beyond cardiovascular disease, our observations have much broader relevance because inflammation is causally implicated in many disease processes [1–8]. We observed that elevated CRP was common across diseases, and in disease- or multimorbidity-stratified analyses it was almost uniformly associated with greater IRR for infection death than for cardiovascular death. We have previously shown that multimorbidity and some morbidities (class 3 obesity, hypertension, chronic respiratory disease, chronic kidney disease, psychiatric disease, chronic inflammatory, and autoimmune rheumatological disease), along with advancing age and increasing SED, were associated with greater risk of infection death than other causes of death [18]. Because elevated CRP was still associated with greater risk of infection death than other causes of death in these subgroups, it is possible that the combination of elevated CRP and these diseases identifies people particularly predisposed to infection death.

An important question arising from our observations is which factors mediate the association between systemic inflammation and infection death. Because elevated CRP was more strongly associated with infection death than cardiovascular or other causes of death in all but 1 of the morbidities we studied, a common mechanism (or mechanisms) seems the most plausible explanation. One possibility is that elevated CRP is a marker of frailty and reduced physiological reserve, and the substantial reduction in IRR between crude and age-sex adjusted models (Supplementary Table 7) may support this possibility. However, the persistent association between elevated CRP and infection death in our “fully adjusted” models suggests that factors beyond frailty and comorbidity are relevant. Another possibility is that elevated CRP is a biomarker of more broadly perturbed immune responses, as observed with aging, and characterized by persistent systemic inflammation and impaired adaptive immune responses to pathogens and vaccines [26–28]. It is notable that recent data have suggested that clonal hematopoiesis of indeterminate potential, a disorder arising from somatic mutations that promote overrepresentation of proinflammatory myeloid clones (and elevated CRP) [29, 30], is associated with increased risk of diverse infections [31]. Therefore, it will be important for future research to better profile the immune milieu in at risk populations, both in periods of usual health and during episodes of infection. These data may help to identify elements of the immune response whose perturbation may predispose to infection, which may act as a useful biomarker and even define safer avenues for anti-inflammatory therapy or strategies to reduce infection risk.

It is also important to interpret our work in the context of some limitations. First, the observational design precludes us from inferring causality in the association between systemic inflammation and infection death. Second, we have no data on the use of immunosuppressive anti-inflammatory therapies beyond the point of study enrollment, or indeed the lifetime doses of these. This is important because some people with undiagnosed inflammatory disorders, either at baseline or emerging during follow-up, may have later commenced these therapies, which are known to increase the risk of infection death. Moreover, lifetime dose of some immunosuppressive agents is associated with increased risk of infection and cardiovascular events [16, 32]. Hence, our adjusted IRR data may overestimate the association between elevated CRP and some causes of death. However, a relatively small proportion of the general population is prescribed such therapy, so our inability to account for incident use is unlikely to substantially diminish our estimates. Our analysis is similarly limited by only having access to CRP and covariate data at baseline. Finally, it is important to note that CRP defines only one facet of inflammation and may not be the optimal biomarker to define or understand this issue; hence, future studies should explore other markers.

## CONCLUSIONS

In conclusion, we show that elevated CRP is common in people with diverse chronic diseases and accumulates with multimorbidity. Irrespective of the threshold chosen, CRP defines a group of people at particularly increased relative risk of infection death. Moreover, this observation persisted in analyses restricted to the majority of comorbidities we studied, indicating that it is broadly relevant. This suggests that using CRP as a biomarker to identify people who may benefit from potent anti-inflammatory therapies also selects a population at increased risk of fatal infection, in keeping with recent clinical trial data in people with atherosclerosis [9]. This raises the question of whether more could be done to prevent infection, for example by improving suboptimal uptake of existing vaccination strategies [16], before commencing anti-inflammatory therapy. In the future, researchers should aim to understand the immune responses to pathogens in people with systemic inflammation, which may help to develop safer anti-inflammatory therapies for chronic disease and target their use to people most likely to obtain net benefit.

## Supplementary Data

Supplementary materials are available at *The Journal of Infectious Diseases* online (http://jid.oxfordjournals.org/). Supplementary materials consist of data provided by the author that are published to benefit the reader. The posted materials are not copyedited. The contents of all supplementary data are the sole responsibility of the authors. Questions or messages regarding errors should be addressed to the author.

## Supplementary Material

jiac186_Supplementary_Data

## Notes

***Disclaimer***. The views expressed are those of the authors and not necessarily those of the National Health Service, the UK National Institute of Health Research (NIHR), or the Department of Health and Social Care.

***Financial support***. This work was funded by the British Heart Foundation (FS/18/44/33792 and FS/12/80/29821). A. W. M. was additionally supported by the NIHR Leeds Biomedical Research Centre  and Diagnostic Evaluation Co-operative  and a NIHR Senior Investigator award. Funding to pay the Open Access publication charges for this article was provided by the British Heart Foundation.

***Potential conflicts of interest***. A. W. M. has previously undertaken consultancy work on behalf of the University of Leeds in relation to giant cell arteritis for Roche, Chugai, GlaxoSmithKline, Sanofi, Regeneron Pharmaceuticals, Astra Zeneca, and Vifor and has received research funding from NIHR and Roche. M. T. K. has received speaker fees from Merck and Novo Nordisk and unrestricted research awards from Medtronic. K. K. W. reports the following: research grants from the British Heart Foundation and NIHR Heart; consultancy work for Medtronic and Cardiac Dimensions; investigator and steering committee membership for studies coordinated by Novartis, Medtronic; participation in data safety and monitoring board or advisory board for Microport and Boehringer Ingelheim; speaker fees from Cardiac Dimensions, Medtronic, Microport, Abbott, Pfizer, Novartis, Boehringer Ingelheim, and BMS; and payment for expert testimony from the UK National Health Service. M. P.-R. is currently employed by Union Chimique Belge Biopharma. All authors have submitted the ICMJE Form for Disclosure of Potential Conflicts of Interest. Conflicts that the editors consider relevant to the content of the manuscript have been disclosed.
